# Prevalence and influencing factors of oral frailty among middle-aged and older patients with chronic kidney disease: a cross-sectional study

**DOI:** 10.3389/fmed.2025.1649113

**Published:** 2025-08-15

**Authors:** Yuelin Wang, Zengli Chen, Qing Tang, Yunlan Jiang, Qianqian Cong, Hong Chen, Lunhui Wu

**Affiliations:** ^1^College of Nursing, Chengdu University of Traditional Chinese Medicine, Chengdu, China; ^2^Department of Nursing, Hospital of Chengdu University of Traditional Chinese Medicine, Chengdu, China; ^3^Department of Nephrology, Sichuan Provincial People's Hospital, School of Medicine, University of Electronic Science and Technology of China, Chengdu, China; ^4^Department of Nephrology, Hospital of Chengdu University of Traditional Chinese Medicine, Chengdu, China

**Keywords:** chronic kidney disease, oral frailty, prevalence, influencing factors, cross-sectional study

## Abstract

**Background:**

Oral health has emerged as a subject of significant public concern. Oral frailty represents the clinical presentation of advanced oral health decline and serves as a significant indicator of systemic frailty. However, current research on oral frailty in patients with chronic kidney disease (CKD) is quite limited.

**Objectives:**

This study aimed to investigate the prevalence and influencing factors of oral frailty among middle-aged and older CKD patients.

**Methods:**

This was a cross-sectional study involving 307 CKD patients from two tertiary general hospitals in Chengdu, Sichuan Province, from November 2024 to March 2025. The Oral Frailty Index-8 (OFI-8), Mini Nutritional Assessment Short-Form (MNA-SF), Revised Piper Fatigue Scale (RPFS) and Hospital Anxiety and Depression Scale (HADS) were used to assess CKD patients. Chi-square tests and logistic regression analyses were used to determine the associated factors of oral frailty among CKD patients.

**Results:**

The prevalence of oral frailty among CKD patients was 61.9% (190/307). In binary logistic regression analysis, marital status, educational level, self-care ability, dentures, dry mouth, and the RPFS-CV score were identified as factors significantly increasing the risk of oral frailty in middle-aged and older hospitalized patients with CKD.

**Conclusion:**

Compared with non-CKD patients, CKD patients demonstrate poorer oral health status, and oral frailty is common among CKD patients. This finding suggests the necessity for healthcare professionals to develop individualized and evidence-based strategies for the prevention and management of oral frailty, with particular emphasis on high-risk populations requiring targeted interventions.

## 1 Introduction

Chronic kidney disease (CKD) is a prevalent, progressive chronic condition (1, 2). Currently, CKD has emerged as a significant global public health challenge, with a global prevalence of 13.4% (3). The prevalence of CKD in China is 8.2%. The high prevalence, substantial disability burden, and considerable healthcare expenditures of this condition impose severe pressures on both society and families, particularly in developing countries such as China (4).

Studies have shown a significant correlation between CKD and oral health, and the interaction between the two creates a vicious cycle. On the one hand, CKD patients frequently experience reduced salivary secretion, resulting in dry oral mucosa and diminished oral self-cleaning ability. Additionally, impaired renal function leads to the systemic accumulation of metabolic waste in oral tissues, thereby increasing the risk of halitosis, dental caries, periodontitis, and other oral health issues (5). On the other hand, poor oral health can increase the burden on the kidneys through bacterial infections and inflammatory responses, accelerating the progression of CKD and even increasing the risk of death in dialysis patients (6).

Oral frailty (OF) is a novel conceptual framework in frailty research, which serves as a significant indicator of systemic frailty. It encompasses structural abnormalities of teeth, muscles, and salivary glands, along with functional impairments, including chewing weakness, swallowing difficulties, reduced food intake, speech impairments, and deterioration of oral hygiene (7). This condition is frequently associated with cognitive dysfunction and systemic health deterioration (8). Li et al. reported a 24.0% prevalence of oral frailty among Chinese elderly individuals, which was generally higher than that in developed countries (9), with poor oral health directly threatening patients’ overall health (10). Previous studies have established significant associations between oral frailty and multiple adverse health outcomes, including cognitive impairment (11), sarcopenia (12), malnutrition (13), and falls (14), all of which substantially impact patients’ long-term quality of life and mortality rates. Furthermore, the associated risks increase significantly with age (15). Therefore, early assessment of oral frailty in CKD patients has two purposes: evaluating oral health status and detecting early signs of physical frailty (16). Early assessment of oral frailty in CKD patients enables timely intervention in oral health issues, thereby reducing the risk of severe complications and adverse health consequences. For example, it helps reduce the spread of infections and inflammatory factors, alleviate systemic inflammation and renal burden, and prevent infectious complications. Additionally, by improving oral function to ensure adequate nutrient intake, it enhances overall physical condition, thereby slowing the progression of CKD and ultimately improving the patient’s overall frailty status.

In recent years, oral frailty has become a critical health concern in clinical practice. However, the majority of studies have focused on oral frailty in the elderly population, with limited research reporting on the prevalence and characteristics of oral frailty in CKD patients. Therefore, this study investigated the prevalence and influencing factors of oral frailty among CKD patients, providing an evidence-based foundation for healthcare professionals to develop targeted prevention strategies, standardized nursing protocols, and optimized follow-up plans. Ultimately, this research aims to effectively enhance oral-related capabilities and improve long-term quality of life in CKD patients.

## 2 Materials and methods

### 2.1 Setting and participants

This cross-sectional study used a convenience sampling approach to recruit participants from two tertiary general hospitals in Chengdu, Sichuan Province, from November 2024 to March 2025. The inclusion criteria were as follows: ① age ≥18 years; ② confirmed CKD diagnosis according to established clinical criteria, encompassing stages 1–5 (17); ③ intact cognitive function with normal intellectual capacity and unimpaired verbal communication skills and capable of completing the questionnaire independently or with researcher assistance; and ④ provision of informed consent for voluntary participation in this study. The exclusion criteria were as follows: ① a history of severe mental illness or significant cognitive impairment; inability to complete the questionnaire; and ② severe cardiac, cerebral, or hepatic dysfunction or acute exacerbation of critical illness.

### 2.2 Measurements

#### 2.2.1 Demographic and clinical characteristics questionnaire

On the basis of a comprehensive literature review and consultation with domain experts, the research team developed a self-designed questionnaire on demographic and clinical characteristics. The form included demographic characteristics, disease-related information, and biochemical indicators: ① demographic characteristics: age, sex, marital status, educational level, family monthly income, residential area, and body mass index (BMI); ② disease-related information: CKD stage, disease duration, comorbidities, dentures, dry mouth, etc.; and ③ biochemical indicators: hemoglobin, albumin, serum calcium, serum phosphorus, creatinine, blood urea nitrogen, GFR, etc.

#### 2.2.2 Oral Frailty Index-8

The Oral Frailty Index-8 (OFI-8) scale, developed by Tanaka et al. from the Japanese Dental Association, is a validated tool for assessing oral health-related frailty (18). The scale comprises 5 domains and 8 items: denture use (1 item), swallowing ability (1 item), chewing ability (3 items), oral health-related behaviors (2 items), and social engagement (1 item). The total score ranges from 0 to 11 points, with a score ≥4 indicating oral frailty. After being translated into Chinese, the scale demonstrated good reliability and validity (19), with a test–retest reliability coefficient of 0.786 and a Cronbach’s alpha coefficient of 0.949. Its validity has been thoroughly validated through cross-sectional studies (20). In this study, CKD patients were classified into two groups: the oral frailty group (OFI-8 ≥ 4) and the nonoral frailty group (OFI-8 < 4).

#### 2.2.3 Mini Nutritional Assessment-Short Form

The Mini Nutritional Assessment Short-Form (MNA-SF), developed by Rubenstein et al. (21) as an abbreviated version of the MNA scale, serves as a rapid screening tool for nutritional assessment. It is suitable not only for screening for nutritional risk but also for diagnosing malnutrition, with high sensitivity and specificity. The MNA-SF comprises 6 assessment domains: weight loss, BMI, disease status, mobility, mental illness, and eating or digestive status. With a maximum score of 14 points, the evaluation criteria are as follows: 12–14 points (normal nutritional status), 8–11 points (at risk of malnutrition), and 0–7 points (malnutrition). The scale has demonstrated excellent test–retest reliability, scale reliability, and validity (22).

#### 2.2.4 Revised Piper Fatigue Scale

The Revised Piper Fatigue Scale (RPFS), originally developed by Piper et al. (23), represents the first multidimensional self-assessment tool designed to evaluate subjective fatigue in patients. Its reliability and validity were validated through testing on 382 breast cancer patients, with a Cronbach’s alpha coefficient of 0.970. In 2003, Hong Kong scholars translated it into the RPFS-Chinese Version (RPFS-CV). The scale comprises 24 items, with each item and a total score ranging from 0 to 10 points. Higher scores indicate more severe fatigue symptoms. The Cronbach’s alpha coefficient for the total scale is 0.910 (24).

#### 2.2.5 Hospital Anxiety and Depression Scale

The Hospital Anxiety and Depression Scale (HADS), developed by Zigmond and Snaith (25), is a well-validated screening tool designed to assess emotional distress in general hospital patients. The scale consists of two subscales: anxiety (7 items) and depression (7 items). Each item is scored on a 4–point Likert scale ranging from 0 to 3 points. The scoring criteria are as follows: 0–7 points indicate negative symptoms (no clinically significant anxiety or depression), 8–10 points indicate mild symptoms, 11–14 points indicate moderate symptoms, and 15–21 points indicate severe symptoms (26). The scale was validated in 265 kidney transplant recipients and demonstrated good reliability and validity. The overall Cronbach’s alpha coefficient for the scale was 0.906, with Cronbach’s alpha coefficients of 0.843 and 0.845, respectively (27).

### 2.3 Data collection

Before the questionnaires were distributed, all the investigators underwent systematic training covering scale interpretation, item specifications, response options, and scoring criteria. To ensure data completeness and objectivity, trained investigators provided face-to-face guidance via uniform instructions during questionnaire completion. The participants were expected to complete the questionnaires independently following the investigators’ explanations. However, for patients with extremely low educational levels or visual impairments who found it difficult to complete the questionnaire, investigators used neutral and nonsuggestive question-and-answer formats. The research team screened electronic medical records daily to identify eligible CKD patients on the basis of the inclusion criteria. After patients’ basic information was obtained, they communicated with them to assess their willingness to participate. After basic demographic information was obtained, the investigators assessed participants’ willingness to enroll in the study. Following the informed consent procedures, the participants spent an average of 20–30 min completing the questionnaire.

### 2.4 Ethical considerations

This study was approved by the Ethics Committee of the Affiliated Hospital of Chengdu University of Traditional Chinese Medicine. The ethical approval number of the study is 2024KL-047-01. This survey was in line with the principles outlined in the latest version of the Declaration of Helsinki. All participants voluntarily participated in this study and signed a paper informed consent form at the beginning of the study. All questionnaires in this study were filled out anonymously, ensuring that all data will only be used for data analysis in this study. Data may not be deposited in public repositories without the permission of participants.

### 2.5 Statistical analysis

All the statistical analyses were performed via SPSS software (version 26.0). Continuous variables with a normal distribution are presented as the means ± standard deviations and were compared via independent samples t tests for intergroup differences. Non-normally distributed continuous variables are presented as median (25th, 75th percentile). Comparisons between groups were performed via the Mann–Whitney U test. For categorical or ordinal variables, statistical descriptions are expressed as frequencies and percentages, whereas comparisons between groups were performed via the chi-square test. Variables with statistical significance in the univariate analysis were incorporated into binary logistic regression to identify independent influencing factors for oral frailty in middle-aged and older hospitalized CKD patients. A two-tailed *p* value < 0.05 was considered statistically significant.

## 3 Results

### 3.1 Participant characteristics

A total of 311 questionnaires were distributed, with 307 valid questionnaires returned, yielding a high response rate of 98.7%. A total of 190 (61.9%) patients were in the oral frailty group, and 117 (38.1%) were in the nonoral frailty group. Among all CKD patients, 85 were aged younger than 60 years, and 222 were aged 60 years or older, with an average age of 67.50 ± 10.22 years. The sex distribution included 184 male patients and 123 female patients. The complete demographic characteristics are shown in Table 1.

**Table 1:** Basic characteristics of CKD patients (*n* = 307).

Variable	Category	Participants	Percentage (%)
Age	<60	85	27.7
	≥60	222	72.3
Gender	Male	184	59.9
	Female	123	40.1
Marital status	Married	259	84.4
	Single/divorced/widowed	48	15.6
Educational level	Middle school or below	197	64.2
	High school or above	71	23.1
	Bachelor or above	39	12.7
Family monthly income	<3,000 yuan	142	46.3
	3,000–5,000 yuan	89	29.0
	>5,000 yuan	76	24.8
Residential area	Countryside	34	11.1
	City and town	273	88.9
Self-care ability	Completely dependent	16	5.2
	Partially independent	73	23.8
	Fully independent	218	71.0
Smoking history	Yes	45	14.7
	No	262	85.3
Drinking history	Yes	23	7.5
	No	284	92.5
Dentures	Yes	131	42.7
	No	176	57.3
Dry mouth	Yes	187	60.9
	No	120	39.1
CKD stage	Stage 1–2	28	9.1
	Stage 3–4	109	35.5
	Stage 5	170	55.4
Disease duration	<5 years	164	53.4
	5–10 years	76	24.8
	>10 years	67	21.8
BMI	Normal	156	50.8
	Underweight	23	7.5
	Overweight	105	34.2
	Obesity	23	7.5
Comorbidities	0	8	2.6
	1	40	13.0
	≥2	259	84.4
MNA-SF	12–14 (Normal nutritional status)	71	23.1
	8–11 (At risk of malnutrition)	184	59.9
	0–7 (Malnutrition)	52	16.9
RPFS-CV	0 (No fatigue)	102	33.2
	1–3 (Mild fatigue)	85	27.7
	4–6 (Moderate fatigue)	83	27.0
	7–10 (Severe fatigue)	37	12.1
Hospital Anxiety Scale	0–7 (No anxiety)	257	83.7
	8–10 (Mild anxiety)	34	11.1
	11–14 (Moderate anxiety)	15	4.9
	15–21 (Severe anxiety)	1	0.3
Hospital Depression Scale	0–7 (No depression)	234	76.2
	8–10 (Mild depression)	46	15.0
	11–14 (Moderate depression)	24	7.8
	15–21 (Severe depression)	3	1.0

### 3.2 Results of univariate analysis of influencing factors for oral frailty

The results of the univariate analysis revealed statistically significant differences (*p* < 0.05) between the oral frailty group and the nonoral frailty group in the following variables: age, marital status, educational level, self-care ability, dentures, dry mouth, comorbidities, MNA-SF score, RPFS-CV score, Hospital Depression Scale score, upper limb grip strength, hemoglobin, albumin, and serum calcium. However, there were no statistically significant differences (*p* > 0.05) between the two groups regarding sex, family monthly income, residential area, smoking history, drinking history, CKD stage, disease duration, BMI, Hospital Anxiety Scale score, triceps skin fold (TSF), calf circumference, serum phosphorus, creatinine, blood urea nitrogen, or GFR. The results are shown in Table 2.

**Table 2:** Univariate analysis of oral frailty in CKD patients (*n* = 307).

Variable	Non-OF	OF	*t/Z/χ^2^*	*p*
	(*n* = 117)	(*n* = 190)		
Age			19.021	<0.001
<60	49 (41.9)	36 (18.9)		
≥60	68 (58.1)	154 (81.1)		
Gender			1.367	0.242
Male	75 (64.1)	109 (57.4)		
Female	42 (35.9)	81 (42.6)		
Marital status			13.353	<0.001
Married	110 (94.0)	149 (78.4)		
Single/divorced/widowed	7 (6.0)	41 (21.6)		
Educational level			6.513	0.039
Middle school or below	65 (55.6)	132 (69.5)		
High school or above	32 (27.4)	39 (20.5)		
Bachelor or above	20 (17.1)	19 (10.0)		
Family monthly income			1.300	0.522
<3,000 yuan	50 (42.7)	92 (48.4)		
3,000~5,000 yuan	38 (32.5)	51 (26.8)		
>5,000 yuan	29 (24.8)	47 (24.7)		
Residential area			0.129	0.720
Countryside	12 (10.3)	22 (11.6)		
City and town	105 (89.7)	168 (88.4)		
Self-care ability			16.816	<0.001
Completely dependent	1 (0.9)	15 (7.9)		
Partially independent	18 (15.4)	55 (28.9)		
Fully independent	98 (83.8)	120 (63.2)		
Smoking history			0.080	0.778
Yes	18 (15.4)	27 (14.2)		
No	99 (84.6)	163 (85.8)		
Drinking history			2.825	0.093
Yes	5 (4.3)	18 (9.5)		
No	112 (95.7)	172 (90.5)		
Dentures			61.199	<0.001
Yes	17 (14.5)	114 (60.0)		
No	100 (85.5)	76 (40.0)		
Dry mouth			15.349	<0.001
Yes	55 (47.0)	132 (69.5)		
No	62 (53.0)	58 (30.5)		
CKD stage			2.044	0.360
Stage 1–2	14 (12.0)	14 (7.4)		
Stage 3–4	42 (35.9)	67 (35.3)		
Stage 5	61 (52.1)	109 (57.4)		
Disease duration			1.983	0.371
<5 years	60 (51.3)	104 (54.7)		
5–10 years	34 (29.1)	42 (22.1)		
>10 years	23 (19.7)	44 (23.2)		
BMI			1.278	0.734
Normal	61 (52.1)	95 (50.0)		
Underweight	10 (8.5)	13 (6.8)		
Overweight	36 (30.8)	69 (36.3)		
Obesity	10 (8.5)	13 (6.8)		
Comorbidities			6.289	0.043
0	4 (3.4)	4 (2.1)		
1	22 (18.8)	18 (9.5)		
≥2	91 (77.8)	168 (88.4)		
MNA-SF			8.638	0.013
12–14 (Normal nutritional status)	33 (28.2)	38 (20.0)		
8–11 (At risk of malnutrition)	73 (62.4)	111 (58.4)		
0–7 (Malnutrition)	11 (9.4)	41 (21.6)		
RPFS-CV			28.700	<0.001
0 (No fatigue)	54 (46.2)	48 (25.3)		
1–3 (Mild fatigue)	39 (33.3)	46 (24.2)		
4–6 (Moderate fatigue)	18 (15.4)	65 (34.2)		
7–10 (Severe fatigue)	6 (5.1)	31 (16.3)		
Hospital Anxiety Scale			6.268	0.099
0–7 (No anxiety)	104 (88.9)	153 (80.5)		
8–10 (Mild anxiety)	9 (7.7)	25 (13.2)		
11–14 (Moderate anxiety)	3 (2.6)	12 (6.3)		
15–21 (Severe anxiety)	1 (0.9)	0 (0.0)		
Hospital Depression Scale			8.527	0.036
0–7 (No depression)	98 (83.8)	136 (71.6)		
8–10 (Mild depression)	15 (12.8)	31 (16.3)		
11–14 (Moderate depression)	4 (3.4)	20 (10.5)		
15–21 (Severe depression)	0 (0.0)	3 (1.6)		
Upper limb grip strength (kg)	20.80 (16.60–29.85)	19.70 (14.38–24.33)	−2.717	0.007
TSF (cm)	1.30 (0.90–1.80)	1.30 (1.00–1.90)	−0.772	0.440
Calf circumference (cm)	33.50 (31.00–35.00)	32.00 (30.00–35.00)	−1.902	0.057
Hemoglobin (g/L)	109.59 ± 23.16	102.05 ± 25.14	2.630	0.009
Albumin (g/L)	41.40 (38.00–44.05)	39.30 (34.48–42.13)	−3.948	<0.001
Serum calcium (mmol/L)	2.21 (2.10–2.35)	2.13 (1.99–2.35)	−2.185	0.029
Serum phosphorus (mmol/L)	1.41 (1.13–1.76)	1.39 (1.09–1.70)	−0.809	0.419
Creatinine (mmol/L)	351.50 (132.90–673.65)	368.65 (167.68–620.73)	−0.018	0.986
Blood urea nitrogen (mmol/L)	18.10 (9.21–23.61)	16.88 (10.79–25.12)	−0.694	0.488
GFR	14.60 (6.50–39.25)	11.65 (6.60–32.98)	−0.735	0.462

### 3.3 Results of binary logistic regression analysis of influencing factors for oral frailty

The results of the multivariate logistic regression analysis revealed the independent influencing factors for oral frailty in middle-aged and older hospitalized CKD patients: marital status (OR = 3.508, 95%CI 1.296–9.493, *p* = 0.013), educational level at middle school or below (OR = 4.099, 95%CI 1.603–10.479, *p* = 0.003) and high school or above (OR = 3.122, 95%CI 1.105–8.823, *p* = 0.032), self-care ability (OR = 12.472, 95%CI 1.367–113.782, *p* = 0.025), dentures (OR = 12.098, 95%CI 5.787–25.293, *p* < 0.001), dry mouth (OR = 2.740, 95%CI 1.471–5.103, *p* = 0.001), and RPFS-CV scores (OR = 1.778, 95%CI 1.226–2.576, *p* = 0.002). The complete analysis results are shown in Table 3.

**Table 3:** Binary logistic regression analysis of factors influencing oral frailty in CKD patients (*n* = 307).

Variable	*β*	*SE*	Wald*χ^2^*	*p*	OR	95%CI
Marital status						
Single/divorced/widowed	1.255	0.508	6.104	0.013	3.508	1.296–9.493
Educational level						
Middle school or below	1.411	0.479	8.676	0.003	4.099	1.603–10.479
High school or above	1.139	0.530	4.615	0.032	3.122	1.105–8.823
Self-care ability						
Completely dependent	2.524	1.128	5.005	0.025	12.472	1.367–113.782
Dentures	2.493	0.376	43.901	<0.001	12.098	5.787–25.293
Dry mouth	1.008	0.317	10.084	0.001	2.740	1.471–5.103
RPFS-CV	0.575	0.189	9.227	0.002	1.778	1.226–2.576

The omnibus test for model coefficients yielded *χ^2^* = 137.00, *p* < 0.001; the Hosmer–Lemeshow goodness-of-fit test yielded *χ^2^* = 11.920, *p* = 0.155.

## 4 Discussion

Frailty is a clinical syndrome characterized by a decline in physiological functions across multiple systems, diminished reserve capacity, and reduced stress resistance, representing a “vulnerable period” between health and disease (28). Currently, the concept of frailty is widely applied in studies targeting the elderly population. Although studies have confirmed that frailty occurs across all age groups in patients with CKD and is one of the primary long-term symptoms in this population, nephrologists may have limited awareness of oral frailty in patients and often overlook oral health issues, further increasing the risk of oral frailty in CKD patients (29). A research by Lee et al. assessed frailty in end-stage renal disease patients undergoing hemodialysis using the Fried phenotype or Edmonton Frail Scale, indicating that frailty is an important predictor of all-cause mortality in such patients. In particular, end-stage renal disease patients exhibit a significantly greater prevalence of frailty, with a prevalence 4 to 10 times greater than that of the general population, directly contributing to increased hospitalization and mortality (30). Oral frailty, as both a localized manifestation and a risk signal for systemic frailty, plays a crucial role in mitigating frailty progression among CKD patients. Early intervention for oral frailty in CKD patients is essential for maintaining their quality of life and overall health, given that poor oral health strongly predicts adverse health outcomes (31).

### 4.1 The prevalence of oral frailty among CKD patients

The results of this study revealed that the prevalence of oral frailty among middle-aged and older CKD patients reached 61.9%, which was significantly higher than the 48.7% reported in prior studies on the prevalence of oral frailty among middle-aged and older hospitalized patients in China (32) and higher than the 30.2 and 28.1% prevalence reported in community-dwelling older adults in China (33) and Japan (34). Several factors may contribute to this elevated prevalence. First, CKD patients often lack adequate knowledge about oral health, and there is a shortage or low utilization of regional oral healthcare resources in China, leading to unresolved oral health issues and progressive deterioration of oral hygiene (35). Second, the long-term burden of chronic diseases and adverse effects associated with dialysis lead to complications such as uremic toxin accumulation, impaired nutrient absorption, diminished oral tissue repair capacity, and increased susceptibility to infections (36), while also predisposing patients to a series of oral diseases, including dental calculus, gingivitis, and dental caries (37). All of these factors impose a significant oral health burden. These findings underscore the necessity for healthcare professionals to prioritize oral health education and promotion, conduct regular oral health assessments, and establish follow-up protocols for hospitalized CKD patients to prevent the onset and progression of oral frailty.

### 4.2 The influencing factors of oral frailty among CKD patients

This study indicated that marital status was a significant factor influencing the development of oral frailty in CKD patients. Maintaining a marital relationship not only fosters the adoption of optimal oral hygiene practices but also contributes to the preservation of a positive psychological state in chronic disease patients, thereby increasing their ability to cope with long-term health challenges. Individuals in stable and fulfilling marriages typically benefit from reliable spousal support, which facilitates mutual health behavior monitoring, consequently reducing the risk of oral frailty (38). Additionally, a Japanese study revealed that single or divorced individuals who frequently dined alone presented irregular meal schedules, faster eating speeds, and inadequate chewing, resulting in insufficient exercise of oral functions and an increased risk of oral frailty (39). Therefore, when developing interventional strategies and public health policies concerning oral frailty for CKD patients, it is essential to consider the multifaceted impact of marital status.

This study also revealed that patients with lower educational levels (those with less than a bachelor’s degree) had a greater risk of oral frailty, which is consistent with the findings of Tu et al. (40). Individuals with limited education typically demonstrate reduced awareness of oral health maintenance, frequently engaging in suboptimal oral hygiene practices such as omitting bedtime brushing, insufficient brushing duration, employing horizontal scrubbing techniques, and maintaining a dietary preference for high-sugar soft foods. This population demonstrates minimal proactive engagement in acquiring knowledge regarding oral health management and disease prevention. Furthermore, they tend to misattribute oral frailty as an inevitable consequence of normal aging processes. This misconception ultimately leads to irreversible deterioration of chewing function and swallowing ability in CKD patients, thereby accelerating the progression of oral frailty (41). These findings underscore the imperative for healthcare professionals to prioritize oral frailty screening among CKD patients with limited education. Individualized oral health education programs and functional exercise schemes tailored to varying educational backgrounds are necessary.

The results of this study revealed that impaired self-care ability was an associated factor for oral frailty in CKD patients. When end-stage CKD patients experience a complete loss of self-care ability, they face difficulties in independently completing routine oral cleaning procedures. This functional limitation leads to the accumulation of food debris and bacteria in the oral cavity, thereby increasing the risk of oral infections, dental caries, and other oral health issues (42). Additionally, CKD patients with poor self-care ability have reduced physical activity, unbalanced nutritional intake, and impaired immune function, which further exacerbates oral frailty (43). Therefore, dynamically assessing the self-care ability of CKD patients and assisting family members in helping patients engage in appropriate physical exercise and oral function training can prevent oral issues from progressing to oral frailty.

The RPFS-CV score was significantly correlated with oral frailty in CKD patients. Research by Chen et al. indicated that fatigue results from multifactorial interactions, and its association with oral frailty may stem from shared pathophysiological mechanisms. Specifically, the systemic inflammatory response and metabolic disturbances resulting from uremic toxin accumulation following renal function deterioration in CKD patients invariably contribute to both fatigue and oral functional decline (44). This suggests that a multidisciplinary team could be established in clinical practice to achieve a balance between protecting renal function and promoting oral health while strengthening physical exercise and nutritional support for hospitalized CKD patients, thereby significantly improving their fatigue status and oral frailty (45).

Denture use has been identified as a significant influencing factor for oral frailty in CKD patients, which is consistent with the findings reported by Li et al. in 363 cancer patients undergoing chemotherapy (46). In CKD patients, disturbances in calcium–phosphorus metabolism and impaired nutritional absorption frequently contribute to alveolar bone resorption and gingival atrophy, which subsequently impair both swallowing coordination and chewing ability. Additionally, reduced saliva secretion not only increases the risk of denture dislodgement but also diminishes the natural cleansing capacity of the oral cavity. This dual effect predisposes patients to fungal colonization and the development of denture stomatitis, thereby increasing susceptibility to oral frailty (47). Consequently, healthcare professionals should first assist CKD patients in maintaining optimal natural dentition and provide specialized denture care guidance for patients wearing dentures. Second, they are encouraged to take regular follow-up examinations of their dentures and oral condition and utilize digital platforms to facilitate follow-up and remote consultation services (48).

This study revealed that dry mouth was significantly associated with oral frailty in CKD patients. In healthy individuals, saliva plays an immune defensive role against oral microorganisms by inhibiting fungal adhesion to epithelial cells (49). However, in CKD patients, impaired renal function adversely affects salivary gland physiology, directly leading to disruption of the oral immune barrier and ultimately resulting in adverse health outcomes such as oral frailty (6, 50). Oral frailty is a reversible condition. For CKD patients with dry mouth, a reasonable drinking and eating plan should be arranged to prevent the proliferation of oral bacteria due to dry mouth symptoms and delay the progression of oral frailty.

Moreover, future studies should examine the close association between CKD stage and oral frailty. Researchers should also further explore the impact of comorbidities in CKD patients, particularly diabetes, as independent variables on oral frailty to compensate for the shortcomings of this study. This will provide healthcare professionals with more professionally targeted prevention measures and standardized nursing strategies.

## 5 Limitations

This study has several limitations. First, this was a cross-sectional study, and causal relationships between various influencing factors and oral frailty cannot be directly inferred. Second, constrained by time, personnel, and funding, this study was conducted in only two tertiary hospitals in Chengdu, Sichuan Province, which means the study population is predominantly of Chinese descent, resulting in sampling bias that limits the representativeness of the sample and the generalizability of the study results to global populations. Third, as the questionnaire relied on self-reported data without objective verification, patient responses were highly subjective and potentially distorted by recall bias (memory inaccuracies) and social desirability bias (underreporting undesirable behaviors), limiting data reliability. Fourth, the relatively short study duration and absence of follow-up assessments make it impossible to confirm the long-term effects of these factors on the progression of oral frailty. Consequently, future studies should expand the scope of investigation and conduct a series of large-scale, multicenter longitudinal studies to verify the findings, providing scientific evidence to reduce the prevalence of oral frailty in middle-aged and older hospitalized CKD patients.

## 6 Conclusion

Oral frailty is a serious condition that develops in the later stages of oral health problems and is a specific manifestation of the patient’s general frailty. The results of this study demonstrate that, compared with community-dwelling older adults and general hospitalized middle-aged and older patients, the prevalence of oral frailty among middle-aged and older hospitalized patients with CKD is as high as 61.9%. This condition has made oral frailty a critical health concern that threatens both the quality of life and survival of CKD patients. Marital status, educational level, self-care ability, dentures, dry mouth, and RPFS-CV scores have been identified as the primary influencing factors for oral frailty in CKD patients. These findings underscore the importance of healthcare professionals conducting early screening to identify individuals at high risk for oral frailty among hospitalized CKD patients. Furthermore, tailored oral health interventions should be developed, incorporating both professional oral hygiene guidance and personalized oral function training programs, with particular attention given to the specific disease characteristics and individual needs of CKD patients. Additionally, a strong family support system can significantly improve oral frailty outcomes and promote long-term oral health maintenance. These evidence-based strategies create favorable rehabilitation conditions for CKD patients, effectively preventing or delaying the onset and progression of oral frailty, thereby achieving a significant reduction in its prevalence among hospitalized CKD patients.

## Data Availability

The original contributions presented in the study are included in the article/Supplementary material, further inquiries can be directed to the corresponding author.
